# Enhancing Unmanned Aerial Vehicle Security: A Zero-Knowledge Proof Approach with Zero-Knowledge Succinct Non-Interactive Arguments of Knowledge for Authentication and Location Proof

**DOI:** 10.3390/s24175838

**Published:** 2024-09-08

**Authors:** Athanasios Koulianos, Panagiotis Paraskevopoulos, Antonios Litke, Nikolaos K. Papadakis

**Affiliations:** Infili Technologies, 15572 Athens, Greece; pparaskevopoulos@infili.com (P.P.); alitke@infili.com (A.L.); npapadakis@infili.com (N.K.P.)

**Keywords:** UAV, authentication, privacy, blockchain technology, ethereum, zero-knowledge proof, zk-SNARKs

## Abstract

UAVs are increasingly being used in various domains, from personal and commercial applications to military operations. Ensuring the security and trustworthiness of UAV communications is crucial, and blockchain technology has been explored as a solution. However, privacy remains a challenge, especially in public blockchains. In this work, we propose a novel approach utilizing zero-knowledge proof techniques, specifically zk-SNARKs, which are non-interactive cryptographic proofs. This approach allows UAVs to prove their authenticity or location without disclosing sensitive information. We generated zk-SNARK proofs using the Zokrates tool on a Raspberry Pi, simulating a drone environment, and analyzed power consumption and CPU utilization. The results are promising, especially in the case of larger drones with higher battery capacities. Ethereum was chosen as the public blockchain platform, with smart contracts developed in Solidity and tested on the Sepolia testnet using Remix IDE. This novel proposed approach paves the way for a new path of research in the UAV area.

## 1. Introduction

The use of unmanned aerial vehicles (UAVs), has gained significant attraction in recent years due to their flexibility and mobility enabling them to participate in hazardous environments where human interaction is impossible. The advances in this domain offer solutions for a variety of tasks and operations including search and rescue missions [1], surveillance operations [2], and emergency response situations [3]. Apart from these, UAVs find applications in numerous domains such as agriculture [4], the supply chain [5], or even acting as relays to enhance network connectivity [6]. Despite the attempts that have been made to secure communication in the Internet-of-Drones (IoD) environments, these devices still lack robust technology that safeguards these aspects. Drones are prone to attacks including DoS, spoofing, or even authenticity attacks, and the communication protocols that are widely used such as the Micro Air Vehicle Link (MAVLink) protocol are still vulnerable [7].

To address some of the security attacks that affect drones and set questions on the operations’ success, researchers have focused on securing the existing protocols [8], or proposing new, more secure schemes [9]. Additionally, an increasing number of studies focus on applying solutions such as blockchains [10] to ensure a reliable, trusted, and secure environment in which drones can operate. Blockchains can offer automation in decision-making and support, bringing solutions to problems regarding air congestion, generating flight paths, and handling emergency situations, especially nowadays where the increased number of UAVs affects all these, and additionally requires resilient solutions.

Despite the promise and innovation that blockchains bring to the security of unmanned aerial vehicles (UAVs), several intricate privacy issues remain unresolved [11]. One of the foremost challenges in modern UAV systems is ensuring that the communication between a drone and a ground control station (GCS) is secure and trustworthy. It is essential for the GCS to accurately identify and authenticate the drone it is communicating with. This authentication is critical because, without it, malicious drones could transmit false and misleading information, potentially compromising the entire mission. The risk of such deception underlines the necessity for robust authentication mechanisms, which are pivotal in maintaining the security and reliability of UAV operations.

Moreover, various UAV applications demand that entities, whether the GCS or the UAV, verify specific conditions such as location without compromising sensitive information. For instance, in military missions, disclosing the UAV’s exact coordinates could jeopardize the mission and endanger personnel. In these high-stakes scenarios, the ability to prove location without revealing classified information is crucial for the mission’s success and the safety of involved entities. Similarly, in commercial contexts, while concealing a drone’s location might be impractical, exposing this information could reveal strategic business insights to competitors, leading to significant operational security risks. Therefore, addressing privacy and security concerns is vital for the effective and secure deployment of UAV systems in both military and commercial settings. Ensuring the protection of sensitive data, verifying authenticity, and maintaining communication integrity are fundamental to the successful use of UAV technology [12].

To address the aforementioned privacy concerns, we propose a novel scheme that leverages zk-SNARK [13] (Zero-Knowledge Succinct Non-Interactive Arguments of Knowledge), a non-interactive, zero-knowledge proof algorithm, for authentication and proof of location. This approach allows the UAV to generate proof of its authenticity and communication with a specific GCS, enabling a secure and decentralized authentication process without compromising the overall system’s security [14]. Furthermore, this technique allows UAVs to verify their presence within an operational area without revealing their exact coordinates [15]. By combining zero-knowledge proof with blockchain technology, a tamper-proof and resistant-to-outside-attacks architecture that enables UAVs and operators to exchange information in a private manner can be achieved. The necessary circuits are developed and tested using the Zokrates tool [16], and the smart contracts are deployed and evaluated on the Ethereum Sepolia testnet [17]. The outcome is a complete set of resources, including the source code, testing files, deployment script, and configuration file, designed to guide developers with software development knowledge but no prior experience in smart contracts.

This work’s structure is as follows: Section 2 briefly presents works related to this area. Section 3 is a thorough examination of the pillars of blockchain technology and zero-knowledge proof schemes. Section 4 is an in-depth review of the proposed architecture, and the tools used to implement it. In Section 5, the results of the proposed scheme and its functionality are presented. Finally, in Section 6, a discussion of the proposed architecture is provided, along with an examination of its limitations and suggestions for future research to enhance this work.

## 2. Related Work

The creation of a secure and trusted environment in the realm of unmanned vehicles, especially UAVs, has gained increased popularity nowadays, thus more and more researchers propose various schemes and frameworks, leveraging blockchain to achieve a solution to this problem.

In [18], the UTM-Chain, a lightweight blockchain-based security solution utilizing Hyperledger Fabric, is designed to address security concerns and provide secure and unalterable traffic data exchanges between UAVs and ground control stations. The authors in [19] try to enhance the security of 5G wireless networks by leveraging blockchain-enabled unmanned aerial vehicles (UAVs). They aim to address the dynamic user demands, irregular data and service requests in smart city scenarios while ensuring reliable and secure service delivery. The work proposed in [20] addresses the limitations of current drone-based systems utilized in Search and Rescue (SAR) missions. To overcome these challenges and enhance SAR quality of service (QoS), the proposed solution integrates blockchain and artificial intelligence at the edge into an Internet-of-Drones (IoD) system architecture.

To face the security challenges posed in multi-drone collaboration scenarios, in case Byzantine UAVs are present (a serious threat to consensus achievement), the authors in [21] proposed a blockchain-based framework to ensure secure communications. In [22], smart contracts are proposed to enhance communication in an intelligent UAV swarm and automate the process of formation selection based on the mission’s nature. A blockchain-based IoT platform for autonomous drone operation management is proposed in [23]. The paper presents findings on Drone-Assisted Wireless Communications for 5G and beyond, showcasing the potential of blockchain in facilitating drone operation management.

The authors in [24] propose a blockchain approach for road traffic monitoring in smart cities utilizing UAVs, addressing congestion challenges by leveraging IoD for comprehensive traffic monitoring. In [25], a novel collaborative approach named B-Drone, integrating Hyperledger Fabric and metaheuristic-enabled genetic algorithms for fog node management is introduced. This work addresses the challenges of privacy, security, and preservation in fog-enabled drone-based data management and optimization. B-Drone ensures integrity, transparency, and security in the processing, scheduling, and management of drone data, leading to improved system robustness and efficiency.

A secure and lightweight blockchain-based authentication scheme for UAVs and RSUs is proposed in [26]. The proposed work is evaluated through both informal and formal methods, including Burrows–Abadi–Needham (BAN) logic, the AVISPA simulation tool, and the real-or-random (RoR) model. In [27], a blockchain-based resource trading mechanism (BRTM) and a double auction-based resource trading algorithm (DARA) for multi-UAV edge computing systems is introduced. The proposed approach integrates blockchain technology with double auction theory to enhance the security and fairness of resource exchanges. The interactions between user equipment and unmanned aerial vehicles (UAVs) are modeled as a two-stage Stackelberg game, where a pricing-based incentive strategy is developed. This strategy promotes active involvement from both UEs and UAVs and aims to maximize their combined utilities. The effectiveness of this method is demonstrated through security evaluations and numerical results, showing its superiority over other benchmark approaches.

A lightweight blockchain for UAV authentication and authorization that integrates non-interactive zero-knowledge proof (NIZKP) with a bilinear map is introduced in [28]. This scheme is tested through four different approaches. The first approach ensures user identity unlinkability, the second adds malleability attack resistance with unlinkability, the third ensures sender trackability by the receiver, and the fourth allows a UAV to delegate transaction tracking to GCS without GCS claiming authority, all supported by security proofs for Signature Unforgeability and Unlinkability in Ciphertext (UN-C). A blockchain-based UAV location authentication scheme that uses a distance bounding protocol to establish location proof and ensure UAV position authenticity is proposed in [29]. This framework employs anonymous certificates and zero-knowledge proof for privacy and has been analyzed for security, with evaluations demonstrating its efficiency and feasibility.

Recent advancements in blockchain and zero-knowledge-proof technologies have opened new avenues for enhancing the security and privacy of unmanned aerial vehicle (UAV) systems. An interesting approach for enhancing privacy in identity management in public blockchains is proposed in [30]. The concept of identity management can be extended to UAVs, ensuring that proof of UAV authenticity can be established without compromising security. Additionally, in [31] Hawk, a blockchain model incorporating cryptographic and privacy-preserving smart contracts, demonstrating how zk-SNARKs can enhance the security and privacy of autonomous systems is proposed. These frameworks highlight the potential of zk-SNARKs in creating tamper-proof and resilient architectures for UAV operations, addressing key challenges such as Byzantine UAVs that try to prove their authenticity or provide false information regarding their position.

While numerous authentication schemes for UAVs exist, the proposed zk-SNARK-based approach addresses critical gaps in current methodologies, particularly in balancing security with privacy. To the best of our knowledge, most of the proposed authentication methods often rely on either symmetric or asymmetric cryptography, which, while secure, may not adequately protect sensitive information such as a UAV’s precise location. Additionally, these methods can be vulnerable to various attacks, especially when deployed in decentralized environments like public blockchains, where privacy is paramount.

The core innovation of our approach lies in leveraging zk-SNARKs to allow UAVs to prove their authenticity or demonstrate specific facts (like their location) without revealing any underlying data. This ensures that even in a public blockchain setting, where transactions and interactions are transparent, sensitive information remains confidential. This capability is particularly crucial in scenarios where revealing a UAV’s location could lead to security breaches, such as in military or strategic commercial operations.

## 3. Background

### 3.1. Blockchain Technology

Blockchain technology allows for efficient data management and recording without the need for a traditional centralized control entity. It bears great potential for improving most sectors by enhancing transparency, security, and efficiency [32,33,34]. Be it finance [35], supply chain management [36], healthcare [37], or intellectual property management [38], blockchain is proving to be a pivotal technology in innovations and systems.

Blockchain is essentially a data structure [39,40] that can be used to store information. The core component of blockchains is the block. Blocks consist of two main parts, the header and the body. Each block’s header contains the hash value of the previous block and a pointer to that previous block, forming a linked structure similar to a chain. Additionally, block headers contain timestamps relevant to the creation of these blocks and a nonce that is the value that miners must determine during the mining process to achieve a specific pattern in the block’s hash (for instance, a block’s hash should end with four zeros). Lastly, a block’s header contains the value of the Merkle tree root. Merkle trees are binary trees that encode the blockchain data in a reverse way (from leaves to root), resulting in a hash that can be used to verify transactions without the need for the users to download the entire ledger.

**Figure 1:**
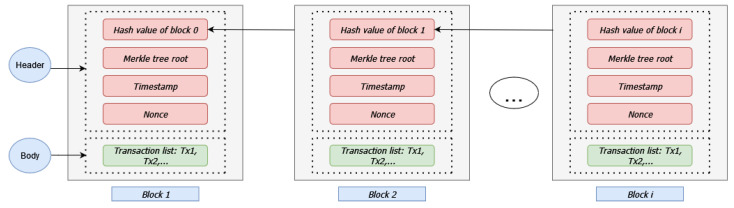
Header and body structure of a block in a typical blockchain.

The data, stored as transactions, are part of the block’s body. These data are essentially the leaf nodes in the aforementioned reverse hashing process in the Merkle tree. Figure 1 illustrates a typical blockchain structure.

From a network perspective, blockchains form a P2P (peer-to-peer) network [41] to submit, forward, and validate transactions. All the nodes in the network share the same privileges. They broadcast the transactions to the neighbor nodes for verification and validation based on their signature. More precisely, each transaction is signed with the sender’s private key and is validated by the network using its public key. However, in an untrusted and decentralized network such as blockchain, there is a need for a mechanism where all participants agree on these transactions (data verification, order, etc.), avoiding the necessity for a central authority. These mechanisms are also known as consensus algorithms [42]. Most known among them are Proof-of-Work (Pow), Proof-of-Stake (PoS), Practical Byzantine Fault Tolerance (PBFT), Proof-of-Authority (PoA), Delegated Proof-of-Stake (DPoS), and many others.

Another core part of blockchain technology is smart contracts [43]. Smart contracts were introduced by Ethereum. They are pieces of code that are activated when some predefined conditions are met. They are mostly used to automate processes and complex transactions. Depending on the platform, they can be developed in languages such as Java, Javascript, Go, and C++. Ethereum has its specific language for writing smart contracts, known as Solidity. A smart contract can be deployed and used between two partners to create transactions between them, supposing that they meet the conditions defined in the contract.

Furthermore, blockchains can be divided into two main categories, public and private. Public blockchains are permissionless blockchains in which anyone can be part of the consensus process and read all the transactions that have been conducted. They usually comprise a high participant number, making them ideal in immutability. However, due to the high participant number, the complexity of the network increases, thus it is less efficient in terms of throughput and latency in comparison with private blockchains. Private blockchains are more efficient since the network is formed by only trusted nodes that have been granted permission to be its members. Even though they exceed in performance, private blockchains are prone to tampering since they are smaller networks and are in a manner more centralized than public blockchains [44].

### 3.2. Zero-Knowledge Proof

Zero-knowledge proof [45,46] is a cryptographic technique that can be used by an entity to prove that a statement is true without revealing any crucial information. zk-SNARKs is a zero-knowledge proof algorithm that requires no interaction between the sender and the verifier [47]. The main components of the zk-SNARK algorithm are the language and relations, the arithmetic circuits, and the quadratic arithmetic programs (QAPs). The language, denoted by L, is a set of statements *x* that an entity wants to prove, while the relations denoted by R are sets constructed by (x,w), where x represents valid statements and w is the corresponding witnesses. A witness *w* is the information that serves as evidence that a particular statement is true. Mathematically, a relation and a language can be described as follows:

$$\begin{array}{cc} R & =\{\left(x,w\right)\;|\;x,w\in {\left\{0,1\right\}}^{*}\},\\ L & =\{x\;|\;\exists \;w:(x,w)\in R\}. \end{array}$$

**Figure 2:**
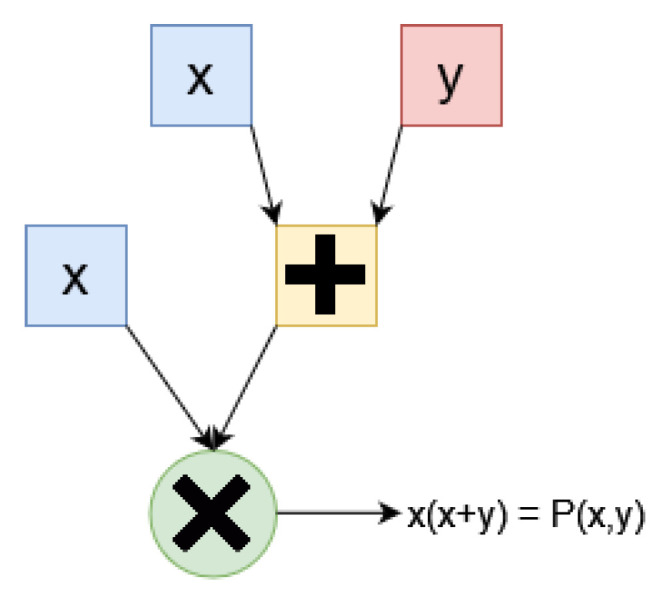
This circuit takes as input x, y and computes the result of x(x+y) using the addition and multiplication gates.

The statements *x* are represented by circuits *C*, which are essentially methods to compute algebraic polynomials. Circuits are similar to boolean circuits, using gates for addition and multiplication, as illustrated in Figure 2.

Finally, QAPs are used to convert these arithmetic circuits into polynomials suitable for the zk-SNARK algorithm through Lagrange interpolation which is a transformation giving a polynomial that passes all (x,y) points that belong in a given set.

The zk-SNARK process can be divided into four main parts, Setup, Keygen, Genproof, and Verproof, which are the polynomial-time algorithms.

Setup$(1^{\lambda })\to Z\;\text{-}$: This algorithm takes as input a security parameter λ and gives as output a set of public parameters $Z\;\text{-}=\{e,p,g_{1},g_{2},G_{1},G_{2},G_{T}\}$, where p is a prime number, e: $G_{1}\times G_{2}\to G_{T}$ is a bilinear map, and where $G_{1}$ and $G_{2}$ are acyclic groups (p-order) with generators $g_{1}$ and $g_{2}$, respectively.

Keygen(C)→(pk,vk): The Keygen algorithm takes as an input the arithmetic circuit and uses the public parameters Z- to generate a pair of keys for proving and verifying the statement: (pk, vk).

Genproof(pk,x,w)→π: The Genproof algorithm takes as input the proof key that was generated by the Keygen algorithm, the statement x, (input of circuit C) and a secret witness *w* (auxiliary input of circuit C), and generates a zero-knowledge proof π based on the relation between the circuit *C*, the statement x and the witness *w*.

Verproof(vk,x,π)→b. The Verproof algorithm takes as input the verification key vk, the statement x, and the proof π and generates as output a binary number based on the proof’s π validity. If the proof is valid, then b=1 or else b=0.

The zk-SNARK scheme satisfies the following properties:

Completeness: If a statement is x∈L, and *w* is a valid witness of x, then the verifier accepts the proof with probability 1.

Pr[Verproof(vk,x,π)→1|Genproof(pk,x,w)→π]=1

Zero knowledge: Without revealing any information regarding the witness *w*, the prover can prove to the verifier that the statement x is true. This can be described mathematically as follows: Let S be a simulator that, given a statement, x∈L and the pk can produce a proof that is indistinguishable from a real proof generated by the prover, without knowing the witness w. Then,

{Genproof(pk,x,w)}≈{S(pk,x)},

where “≈” denotes computational indistinguishability.

Soundness: If a witness w is not valid, then a malicious actor cannot craft a proper proof. If we denote the generated malicious proof with $\tilde{\pi }$, then

Pr[Verproof(vk,x,π)→1]≤ϵ,

where ϵ negligible.

Over the years, several schemes have been proposed for zk-SNARKs. In [48] Pinocchio, a nearly practical implementation of verifiable computation using zk-SNARKs was presented. Pinocchio is one of the earlier zk-SNARK protocols that enables efficient, verifiable computation. It introduced a succinct verification process that is significantly faster and more efficient than previous methods. Pinocchio’s primary contributions include a highly optimized verification algorithm and the use of quadratic arithmetic programs (QAPs) for circuit representation.

Another scheme that was proposed is Marlin. Marlin is a zk-SNARK protocol that enhances efficiency and scalability through a universal and updateable setup, allowing the same setup to be reused across multiple computations. It optimizes polynomial commitment schemes to enable succinct proof of polynomial evaluations, resulting in smaller proof sizes and faster verification times. In [49], a direct comparison between Marlin and other schemes, such as Mal19 and Sonic, showcases the fact that Marlin outperforms in all relevant efficiency parameters (argument size over BN-256 bytes and over BLS12-381 bytes, time complexity for generation, proof verification, etc.). However, Marlin did not manage to perform better than the Groth16 algorithm [50].

Groth16 is the scheme that we used in our work. This scheme is known for its simplicity and efficiency since it outperforms previous schemes by providing shorter proofs and faster verification times. Groth16 can be utilized in blockchain applications due to its compact proof size, efficient verification process, and low time complexity. For all these reasons, we chose this protocol for our proposed UAV proof of authenticity and proof of location schemes.

## 4. Proposed Scheme

In order to mitigate the risk of a malicious drone connecting with the ground control station (GCS) and transferring false data, we propose the following scheme for privacy-preserved operations in UAV systems.

**Figure 3:**
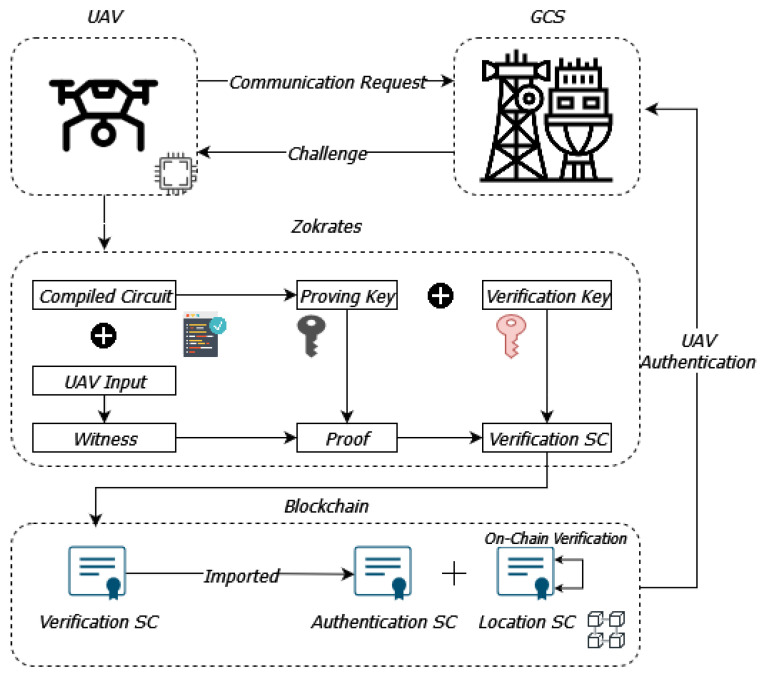
Proposed architecture for private UAV communications utilizing the zk-SNARK algorithm.

Firstly, there are four main participants in such a system: the UAV, the ground control station (GCS), the user who interacts with the UAV through the GCS, and the smart contract (SC) that automates the verification process on-chain (blockchain). The trusted GCS develops and deploys the SC on the Ethereum blockchain. The SC is designed to include the verification process code for the authentication and proof of location of the UAV, as it is produced by the Zokrates tool. A high-level overview of the proposed architecture is illustrated in Figure 3.

The process begins when the UAV sends a communication request to the GCS. In response, the GCS issues a challenge to the UAV, which involves specific input data that the UAV must use to generate proof of its authenticity and location. This challenge-response mechanism ensures that the UAV is actively engaged in the verification process.

Next, the UAV uses the Zokrates tool to compile the circuit required for generating zk-SNARK (Zero-Knowledge Succinct Non-Interactive Arguments of Knowledge). This circuit includes the proving key and verification key, which are essential for the cryptographic operations involved. The UAV inputs the required data, known as the witness, into the compiled circuit to generate proof. This proof is a cryptographic guarantee that the UAV’s location and identity are valid, without revealing the actual sensitive data.

Once the proof is generated, it is sent to the verification smart contract (SC) on the blockchain. The smart contract, which has been pre-deployed by the GCS, includes the necessary logic to verify the proof. This involves using the verification key to check the validity of the proof provided by the UAV. If the proof is valid, the smart contract confirms the UAV’s authentication and location, ensuring that only legitimate UAVs can interact with the GCS and transmit data.

The verification SC imports the other two SCs: the authentication SC and the location SC. The authentication SC is for the on-chain verification of the authenticity of the UAV, and at the same time keeps a list of the already authenticated UAVs, while the location SC handles the verification of the UAV’s geographical location. These contracts utilize the verification logic from the verification SC as was generated by the Zokrates tool.

By leveraging the blockchain for on-chain verification, the proposed scheme ensures that the UAV authentication process is transparent, tamper-proof, and resistant to external attacks. This architecture not only enhances the security and reliability of UAV operations but also preserves the privacy of sensitive information. The use of zk-SNARKs ensures that the UAV can prove its identity and location without disclosing actual data, thereby protecting the privacy of the UAV’s operations and the user’s interactions with the system.

### 4.1. Authentication

The authentication process for the UAVs can be described in the following steps.

(i) Let G be an elliptic curve group defined by the equation $y^{2}=x^{3}+4$, over the finite field $F_{p}$, where $p=2^{128}-19$. The UAV generates the private key $s_{k}$ as a random number in $Z_{p}$. The corresponding public key is calculated using the generator *g* of the elliptic curve group G: $p_{k}=g^{s_{k}}$. The algorithm that is used for the key generation process is based on the BN-128 (Barreto–Naehrig) elliptic curve [50].
(ii) The UAV stores its generated public key $p_{k}$ in the blockchain B as a transaction. The GCS verifies this transaction and stores the $p_{k}$ in its database, ensuring that only trusted UAVs can participate.
(iii) When a UAV requests a connection to communicate with the GCS, the GCS generates a challenge *c* demanding the UAV to prove that it possesses the corresponding secret key $s_{k}$ based on the registered public $p_{k}$. The challenge then is sent to the UAV.
(iv) The UAV generates a proof π for the statement based on the pseudocode in Algorithm 1. The circuit is compiled and the witness is calculated based on the UAV’s input. The proof is then generated based on this witness and the drone’s secret key and is stored in the blockchain along with the verification key (verifier.sol): $UAV\to (v_{k},\pi )\in B$. The Zokrates process for generating the proof is presented in more detail in Figure 3.
**Algorithm 1** Zokrates circuit pseudocode for UAV authentication1:**function** UAVAuthentication($s_{k}$, $p_{k}$) → bool2:      **Initialize:** generator point *g*3:      public key $P_{k}\leftarrow \mathrm{ECC}\_\mathrm{multiplication}\left(g,s_{k}\right)$4:      **bool** valid $\leftarrow (P_{k}==p_{k})$5:      **return** valid6:**end function**
(v) The proof verification for the UAV authenticity happens on-chain through the verification smart contract (SC). If the proof is valid, $Ver(\pi ,p_{k})\to 1$, then the UAV is authenticated and the list with authenticated drones is updated to include the last UAV that proved the challenge. The SC used for the UAV authentication is illustrated by Algorithm 2.

**Algorithm 2** Smart contract for UAV authentication
 1:Import: verifier.sol 2:Verifier verifier 3:mapping(address → bool) authUAVs 4:**function** verProof($a,b,c,input,p_{k}$) → bool 5:      bool valid verifier.verifyProof(a,b,c,input) 6:      **if** valid **then** 7:            authUAVs[$p_{k}$]← 1 8:      **end if** 9:      **return** valid10:
**end function**



### 4.2. Proof of Location

After the drone has been authenticated, it can prove that it has already been in a specific area without revealing its exact coordinates. The drone retrieves its coordinates from the GPS. Then, it generates a proof that its coordinates lie between the predefined boundaries using the Zokrates circuit provided by Algorithm 3. Finally, the proof is submitted to the blockchain as in the authentication case. The verification happens on-chain through the SC. A pre-authenticated drone can now prove that is in a specific location without revealing its coordinates. If the proof is valid and the GCS verifies it, it can ensure that the UAV operates inside the specified area.
**Algorithm 3** Zokrates circuit pseudocode for the UAV operation area1:**Input**: *x*, *y*, *z*2:**Input**: x1, y1, z1, x2, y2, z23:**bool** InX←(x≥x1andx≤x2)4:**bool** InY←(y≥y1andy≤y2)5:**bool** InZ←(z≥z1andz≤z2)6:**bool** valid← (InX and InY and InZ)7:**return** valid▹ UAV’s coordinates (private) ▹ Bounding box coordinates (public)     

The following function presented in Algorithm 4 is part of the SC and is used to verify the location proof that was submitted by the UAV on the blockchain. This functionality could prove invaluable in scenarios such as confidential military missions or commercial drone operations where revealing their exact location is not permissible.

Here, *a*, *b*, and *c* are the proof elements used in the construction of zk-SNARK for Groth16 [50]; this is the key part of the proof so that both its integrity and zero-knowledge property can hold. More precisely, commitment element *a* is a combination of the secret parameter α, the value of polynomial in secret point *s* and a randomization factor, while *b* is built similarly using a different secret parameter β and another randomization factor element. Finally, *c* is a more complex element since it is calculated from a combination of mid-polynomial evaluations of these polynomials to ensure the entire proof’s consistency. A pairing equation that utilizes these elements is used by the verifier to confirm the proof’s validity without revealing any information about the underlying witness.
**Algorithm 4** Pseudocode for the proveLocation function1:**function** proveLocation(*a*, *b*, *c*, input)2:      **Require:** authUAVs[msg.sender] is true 3:      **if** authUAVs[msg.sender] == 0 **then**4:            **Revert with error** “UAV not authenticated”5:      **end if**6:      valid ←verifier.verifyProof(a,b,c,input)7:      **return** valid8:**end function** ▹ Ensure the UAV is authenticated   ▹ Verify the zk-SNARK proof  

**Figure 4:**
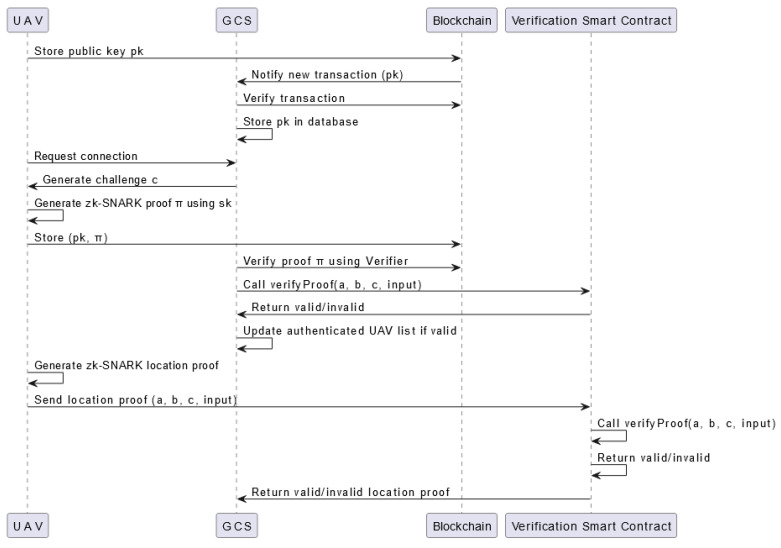
A comprehensive UML sequence diagram illustrating the entire process of UAV authentication and operation area verification using the zk-SNARK algorithm.

A UML diagram of the proposed scheme that describes in detail the process of UAV authentication and the location proof is illustrated in Figure 4.

## 5. Evaluation

The proposed smart contracts were evaluated through the Sepolia test network, which is an Ethereum testnet that uses PoA as a consensus mechanism. The smart contracts were compiled and run through the remix IDE platform [51]. The Zokrates version which upon the proposed cicruits were built and the proofs were generated was 0.8.4 in the Ubuntu 22.04 LTS operating system. As was mentioned in Section 3.2, the proof system that was used for the zk-SNARK proof construction is the Groth16 system, which is a scheme based on pairing-friendly elliptic curves, such as the Barreto–Naehrig 128-bit curve (in our case). Groth16 was chosen because of its smaller proof sizes, constant-time verification, and better performance [52,53] over the Pinocchio, Marlin, GM17, and other schemes.

**Figure 5:**
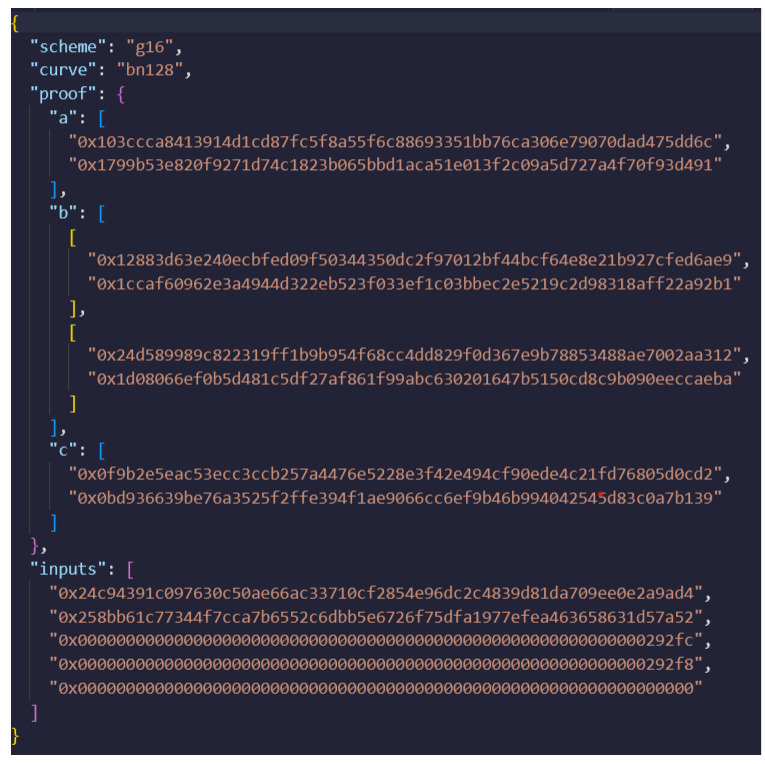
Proof file, as was generated by Zokrates using the Groth16 scheme.

The first step is to compile the proposed circuit for the UAV authentication that it is illustrated in Algorithm 1. Zokrates compiles the .zok circuit file and generates an output file which includes the corresponding polynomials. After the successful compilation of the circuit, the setup step follows, where the polynomials from the output file are used to generate the proving (PK) and verification key (VK) pair, using the Groth16 scheme. Furthermore, the witness is computed based on the user’s private key, a public statement, and the polynomials that were generated during the compilation process of the circuit. This witness is then used along with the PK to generate the corresponding proof. The JSON representation of this proof is illustrated in Figure 5.

In Figure 5, the scheme labeled as “g16” refers to the Groth16 zk-SNARK system, while the curve mentioned, “bn128”, refers to the Barreto–Naehrig curve with a 128-bit security level. Furthermore, the *a*, *b*, and *c* proof elements are presented in this JSON in hexadecimal format. In addition to these proof elements, the JSON file includes an “inputs” section, which lists the public inputs to the zk-SNARK. These inputs are crucial because they typically encode information about the computation or statement being proven; thus, they allow the verifier to confirm the proof’s validity without revealing the prover’s private data (the witness). The structure of the proof—being both compact and efficient—makes it ideal for submission to a smart contract for on-chain verification, especially in platforms like Ethereum, where minimizing gas costs is essential.

The proof generated by the UAV can be verified using the corresponding verification key (VK). This process involves submitting the proof to a smart contract designed to perform the verification on the Ethereum blockchain. If the verification is successful, the contract will return a “PASS” status, indicating that the proof is valid and that the UAV can be authenticated. If the verification fails, the contract will return a “FAILED” status, meaning that the proof is invalid and the UAV’s identity cannot be confirmed.

**Figure 6:**
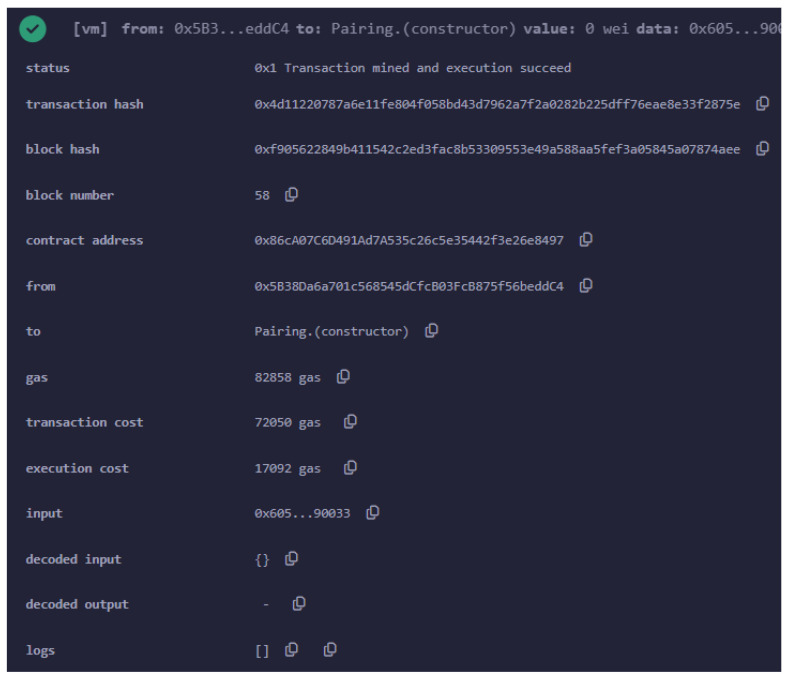
Verification smart contract deployed on Sepolia testnet.

The smart contract responsible for this verification is referred to as verifier.sol.Once generated, this contract is deployed to the Ethereum blockchain. As illustrated in Figure 6, the contract was deployed on the Sepolia testnet, with the contract address being 0x86c...97. The UAV or user deploying this contract is associated with the Ethereum address 0x5B3...C4.

The image captures key details of the deployment transaction. The status field shows that the transaction was successfully mined and executed, confirming that the smart contract was successfully deployed. The transaction hash 0xd411...3f879e and block hash 0xf905...d87a9ee are unique identifiers for this specific transaction and the block in which it was included, respectively.

Finally, deploying a smart contract on Ethereum involves a cost, measured in gas, which is the unit of computational work in Ethereum. The cost for deploying this verification smart contract was 82,858 gas units. The cost of gas fluctuates depending on network conditions, and in this case, an average gas price of GWEI 6.195 was assumed. Given this price, and knowing that GWEI 1 equals ETH $10^{-9}$, the total deployment cost of this smart contract is approximately ETH 0.0005095767. Based on the exchange rate at that time (June 2024), this amount corresponds to around USD 1.78.

**Figure 7:**
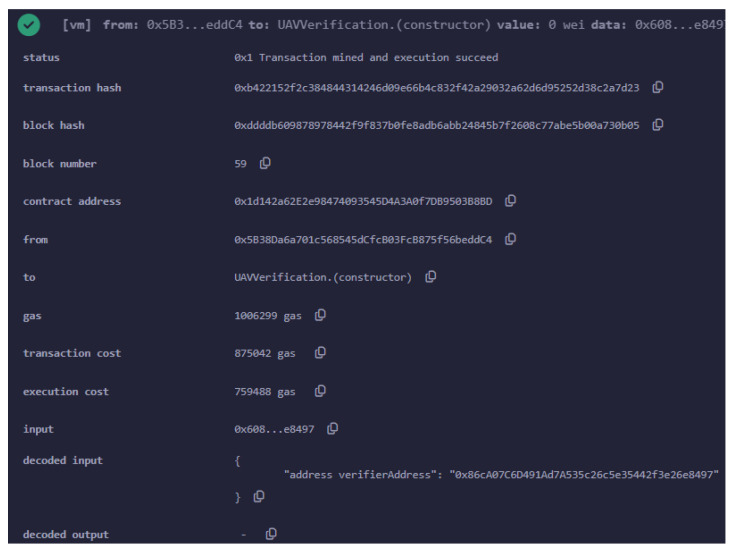
The main smart contract including the verifier.sol, as given by the Algorithm 2, deployed on the Sepolia testnet.

Figure 7 illustrates the deployment of the main smart contract, which incorporates the verifier.sol contract, on the Ethereum Sepolia testnet. The main smart contract is presented by Algorithm 2. This contract facilitates the verification of zk-SNARK proofs, crucial for the authentication process.

The transaction was successfully mined, as indicated by the status, while the transaction hash 0x211...a2b uniquely identifies this deployment transaction. The block hash 0x87f...d0e and block number indicate the specific block in the blockchain where this transaction was recorded, and is uniquely identified by the transaction hash.

The contract was deployed to the Ethereum address 0x1d3...0b03, which now serves as the address where this smart contract resides on the Sepolia testnet. The deployment was initiated from the address 0x5b3...c4, the same address that was involved in deploying the initial verifier.sol contract.

In terms of cost, deploying this smart contract consumed a total of 1,006,299 gas units. Given an average gas price, this amount translates to approximately ETH 0.00618873885, which was worth about USD 21.96 at the time of deployment.

**Figure 8:**
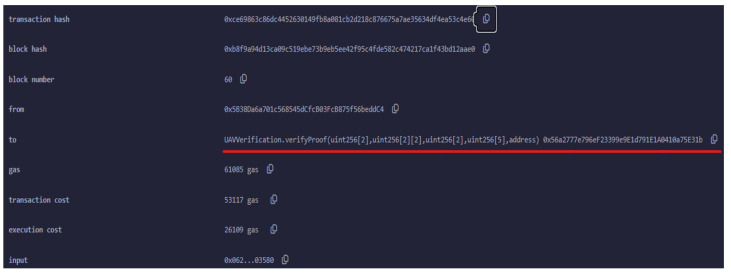
Verification transaction for the UAV authentication. The red line outlines a call to the verifyProof function within the UAVVerification smart contract.

Figure 8 details the verification transaction for UAV authentication, highlighting the final step in the process where the UAV’s proof is submitted to the smart contract for verification. After the successful deployment of the smart contract, the UAV generates a zk-SNARK proof to authenticate itself to the ground control station (GCS). This proof is sent as part of a transaction invoking the verifyProof() function of the smart contract. The verifyProof() function is specifically designed to handle the verification of zk-SNARK proofs. In this context, it ensures that the UAV’s claims (such as its identity or the validity of certain conditions such as location) are correct without revealing any sensitive information.

The transaction shown in Figure 8 was successfully executed, as indicated by the status. The transaction hash 0xcc6...de3e and block hash 0x8f6...83a9e uniquely identify this specific transaction within the blockchain, while the block number places it within the sequence of blockchain events.

The key focus here is on the gas used for the verification process. The transaction consumed 61,085 gas units, with a transaction cost of 53,117 gas units dedicated to the actual verification operation. This cost reflects the computational resources required to execute the zk-SNARK verification on the Ethereum network. At the time of the transaction, this amounted to ETH 0.000329059815, or approximately USD 1.16.

**Figure 9:**
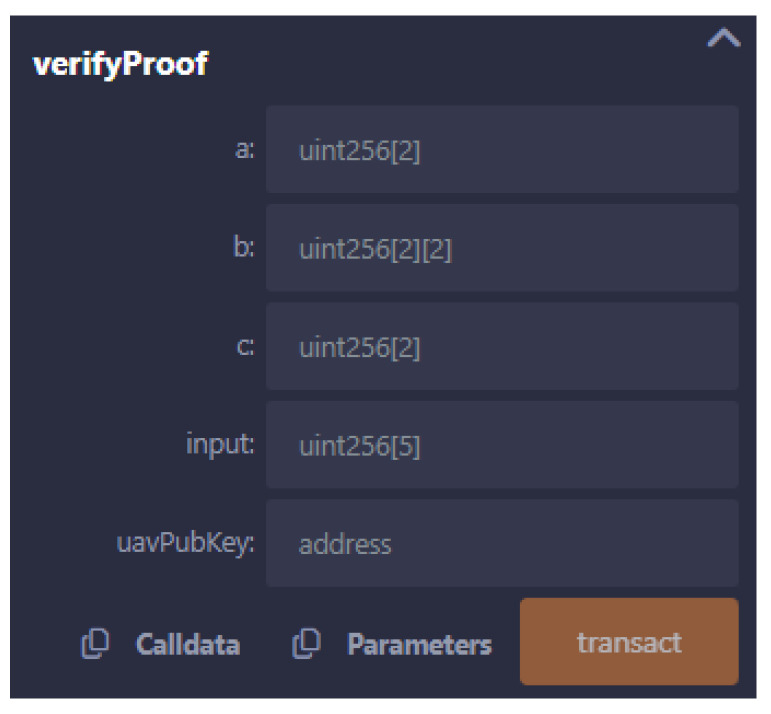
Smart contract’s interface for UAV authentication.

Figure 9 presents the interface of the smart contract used for UAV authentication. This interface is crucial for the GCS when verifying the identity and authenticity of a UAV. The interface requires the input of several parameters, a, b, and c, which are the proof elements generated by the zk-SNARK proof (Figure 5), as well as the input values and public key of the UAV uavPubKey. The elements a, b, and c are the cryptographic components that encapsulate the proof, ensuring that the UAV’s claims can be verified without revealing any sensitive underlying information. The input field contains the public inputs that the verifier uses to confirm the proof’s validity, while the uavPubKey parameter is the public key associated with the UAV. This key helps to ensure that the proof is tied to a specific UAV, preventing impersonation or other forms of fraud.

Once the GCS inputs these values into the interface and initiates the transaction by clicking the “transact” button, the smart contract will execute the verifyProof() function. This function will process the provided proof against the stored verification key (VK) and determine whether the UAV’s proof is valid on-chain. If successful, the UAV’s authenticity is confirmed, and the GCS can securely interact with the UAV, confident in its identity.

This interface also highlights the flexibility of zk-SNARK-based authentication systems. For instance, the same interface structure can be adapted for other verification tasks, such as proving that a UAV is within a specific area without disclosing its exact location. In such cases, a slightly modified circuit—compiled (Algorithm 3) similarly to the authentication circuit using ZoKrates—can produce a proof that only requires the parameters *a*, *b*, *c*, and input. The public key uavPubKey may no longer be necessary if the UAV has already been authenticated, streamlining the process for subsequent verifications.

By utilizing this interface, the GCS can efficiently manage UAVs, verifying their identity or confirming their location with minimal overhead. The use of zk-SNARKs ensures that these verifications are both secure and privacy-preserving, making it possible to deploy UAVs in sensitive operations without compromising their confidentiality or security.

**Figure 10:**
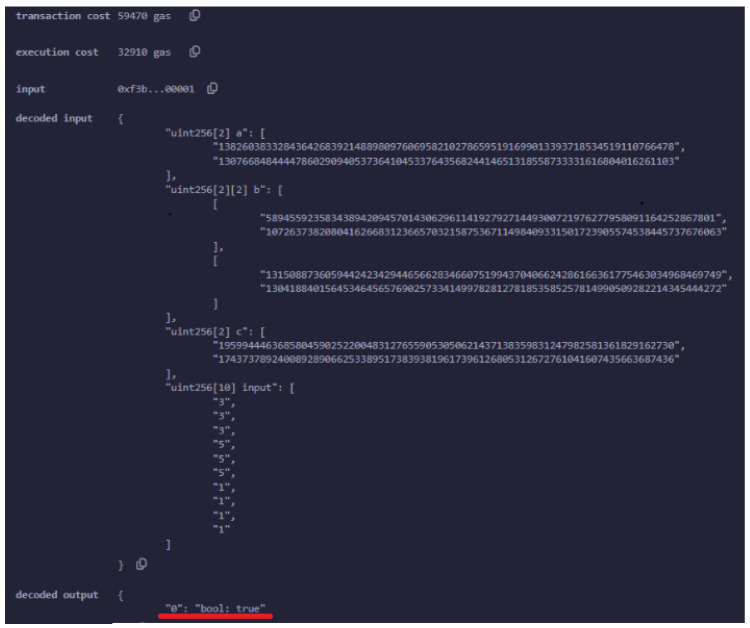
An example where the UAV generates a valid proof for its location. The boolean value true returned by the smart contract, as highlighted by the red line in the figure, confirms the validity of the proof.

If the proof is valid, and the UAV is inside the bounds, then the output of the transaction is true, as illustrated in Figure 10.

In the event that the UAV is not located within the designated area or if the proof of its location is found to be invalid, the system will return a false value.

**Figure 11:**
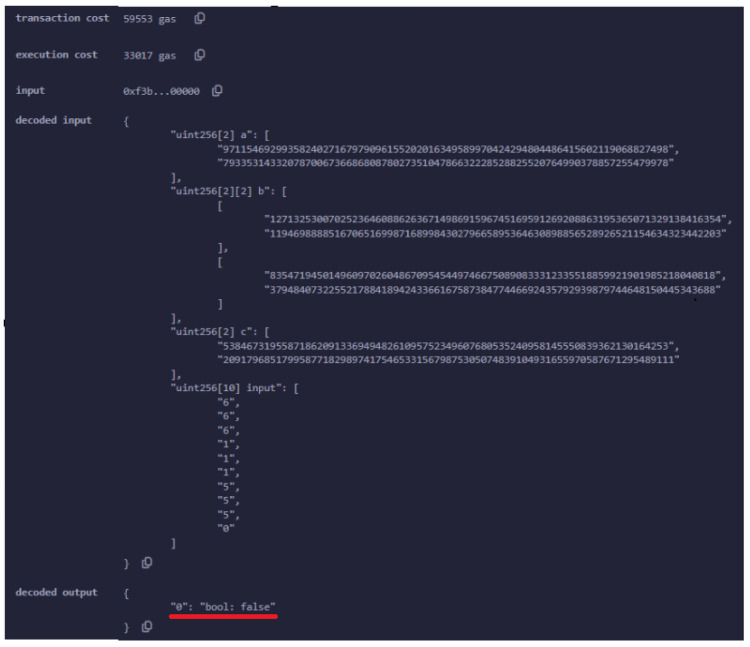
An example where the proof is not valid. The boolean value false returned by the smart contract, as highlighted by the red line in the figure, indicates that the proof is invalid.

It is important to note that for the verification process to be considered valid, the UAV must have been properly authenticated prior to generating the proof. This prerequisite ensures that only legitimate UAVs, which have already been authenticated, are capable of participating in the verification process, thereby maintaining the system’s security and integrity (Figure 11).

**Figure 12:**
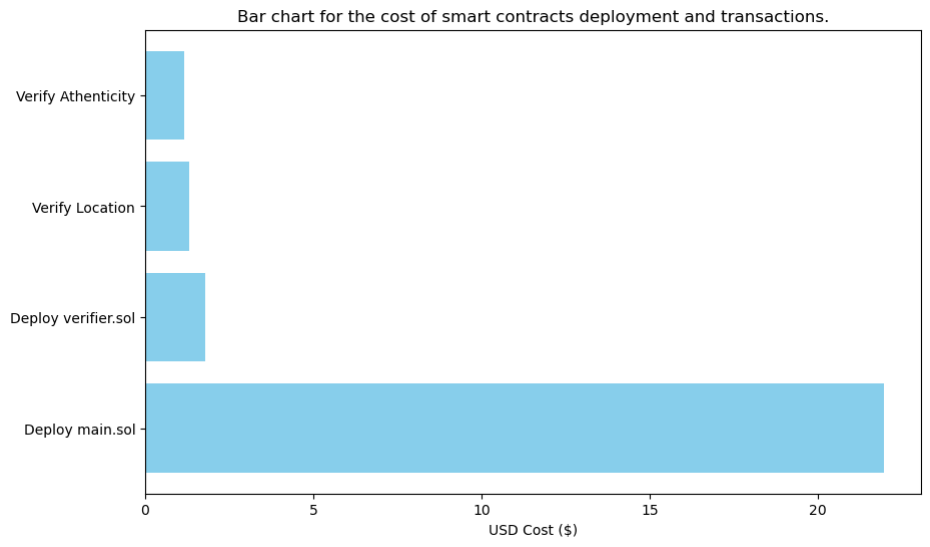
Cost for deploying and interacting with the proposed SCs in Ethereum Sepolia testnet.

The financial cost associated with conducting a transaction to verify whether the UAV is within the specified area or not is approximately 59,470 gas units. This amount of gas translates to roughly ETH 0.00036841665, which is approximately USD 1.3. These costs are significant, particularly in scenarios involving multiple UAVs or frequent verification requests, as they can add up quickly and impact the overall budget for UAV operations. The bar graph presented in Figure 12 provides a visual summary of the costs in USD associated with deploying and interacting with the smart contracts that were presented before. This graph is crucial for understanding the financial implications of utilizing blockchain technology in zk-SNARK-based UAV operations, as it breaks down the various costs associated with different stages of smart contract deployment and interaction. The costs illustrated in this graph are vital for strategic planning and decision-making within organizations, considering the implementation of blockchain-based solutions for UAV authentication and area verification.

To provide a better understanding of the energy consumption, which is crucial in the cryptographic techniques that are applied in UAV operations, we benchmarked the power consumption on an isolated docker container that runs the Zokrated tool, in our Raspberry Pi. High power consumption could lead to high operational costs, especially under heavy computation. This could jeopardize UAV missions, especially in the military context. Therefore, it is important to measure the power consumption when developing and implementing cryptographic schemes, especially where blockchain is used in authentication and verification steps.

**Figure 13:**
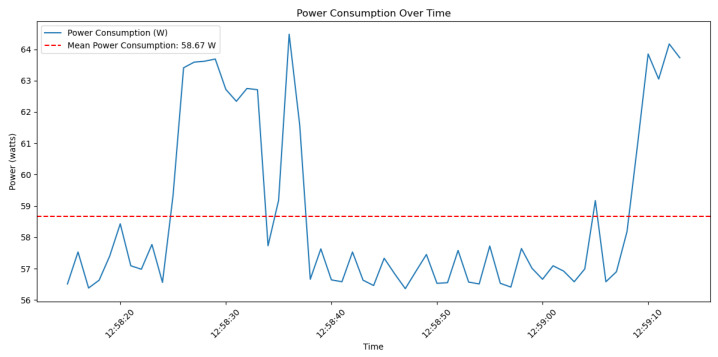
Power consumption over time for CPU operations in zk-SNARKs.

The graph in Figure 13 shows the power usage of the CPU while performing elliptic curve operations, which are fundamental in the proof generation process of zk-SNARKs. The power consumption values in the graph vary over time due to variations in the CPU load levels associated with different stages of the zk-SNARK computation. These variations are a result of several factors specific to zk-SNARKs. The operations involved in constructing zk-SNARK proofs, such as arithmetic circuit evaluation and elliptic curve multiplications, vary in computational complexity, leading to corresponding fluctuations in power consumption. During the proof generation phase, more intensive computations occur, causing higher power usage, while the verifier exporting phase may consume less power.

**Figure 14:**
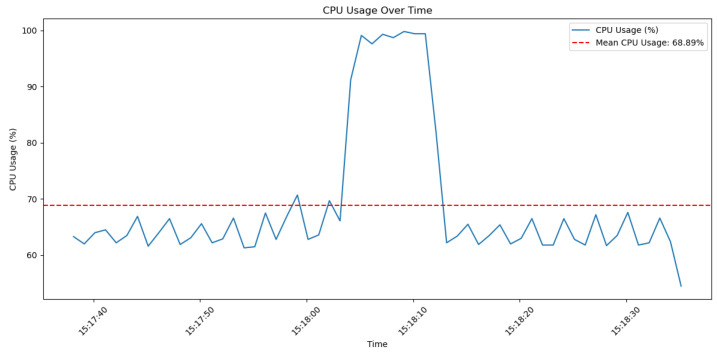
CPU utilization over time for CPU operations in zk-SNARKS.

Additionally, the graph in Figure 14 demonstrates the CPU utilization over time while performing the same elliptic curve operations, which are integral to zk-SNARK proof generation. The fluctuations in CPU usage highlight the varying computational demands during different stages of the zk-SNARK operations.

This variability is due to the different complexities of the operations being performed. Certain phases of zk-SNARK generation require more computational resources, leading to spikes in CPU usage, while other phases may be less demanding. The red dashed line indicates the mean CPU usage during the benchmarking period, which is approximately 68.89%. Despite the peaks and troughs, the average CPU usage remains relatively high, reflecting the intensive nature of zk-SNARK computations.

## 6. Discussion

In our work, we propose a novel scheme that leverages the zk-SNARK protocol to enable UAVs to prove their authenticity and location. Using the Zokrates tool, we compiled the circuits and generated the proofs. Our results suggest that while this approach is power-intensive and thus better suited for drones with higher battery capacities, it holds significant promise. Additionally, we optimized the smart contracts to be as lightweight as possible to minimize gas fees. These contracts were evaluated on the Sepolia testnet. Our proposed solution is versatile, offering potential applications not only for authentication but also for geo-fencing and related use cases. By incorporating zk-SNARKs, UAVs can verify their adherence to defined geographical boundaries without disclosing their precise location data (stay within certain areas or avoid restricted zones).

To the best of our knowledge, the idea of using zero-knowledge proof protocols such as the zk-SNARKs that we used in this study for authentication and proof of locations has so far not been considered in the literature. The method explored in this paper is distinctive, particularly among existing published research, as evidenced by the lack of schemes demonstrating this capability. However, there are still some limitations that we should be aware of and that we further analyze below.

### 6.1. Security and Privacy Analysis

The approach proposed in this work offers solutions to several critical issues present in traditional systems by combining zk-SNARKs with blockchain technology, providing robust protection against a wide range of security threats while inheriting the benefits of both technologies.

One of the key benefits of zk-SNARKs is their ability to allow a UAV to demonstrate knowledge of specific information, such as identity or location, without revealing the information itself. This zero-knowledge property ensures that sensitive data remain confidential throughout the authentication and verification process. This approach not only preserves the confidentiality of sensitive data but also maintains the integrity of the information being processed. Even if communication between UAVs and the GCS is intercepted, the zk-SNARK proofs provide no exploitable information to the attacker, thereby securing critical data like the UAV’s operational parameters or mission details.

Additionally, the incorporation of blockchain technology enhances security by decentralizing the verification process. Traditional systems often rely on centralized servers for verification, creating single points of failure vulnerable to attacks. In contrast, blockchain distributes the verification process across multiple nodes in the network, reducing the risk of attacks targeting a single entity. This decentralized approach ensures that the system’s integrity does not depend on any one node or server.

Furthermore, the blockchain’s immutable ledger guarantees that once a zk-SNARK proof is verified, the record of that verification cannot be altered or tampered with. Every transaction, including proof verifications, is permanently recorded across all nodes, making it extremely difficult for any malicious actor to modify or falsify the records without detection. This tamper-resistant feature of the blockchain provides robust defense against attempts to alter verification results or compromise UAV authenticity.

The combination of zk-SNARKs and blockchain technology also effectively mitigates common attack vectors such as replay attacks and man-in-the-middle attacks. Each zk-SNARK proof is uniquely generated for a specific transaction and timestamp, ensuring that the proof cannot be reused by an attacker in a different context. The blockchain’s time-ordered, immutable structure further reinforces this protection by recording each verification transparently and permanently, ensuring that every action can be traced back to its origin. This approach makes it impossible for attackers to reuse or manipulate old proofs.

Moreover, the cryptographic robustness of zk-SNARKs, coupled with the decentralized nature of blockchain, guards against MITM attacks. Even if an attacker manages to intercept the communication, they cannot alter the zk-SNARK proof or inject fraudulent data without being detected by the blockchain’s consensus mechanism, which requires agreement across multiple nodes before any transaction is validated. This makes it extremely difficult for an attacker to compromise the system.

Ensuring the authenticity of UAVs and preventing impersonation is another critical aspect of the proposed scheme. By directly linking UAV identity to zk-SNARK proofs, the system guarantees that only legitimate UAVs can be authenticated. This cryptographic binding of identity to proof eliminates the possibility of impersonation, as the zk-SNARK proof can only be generated by a UAV with the corresponding private key.

The blockchain further strengthens this security by immutably recording each verification, ensuring that once a UAV is authenticated, its identity cannot be tampered with or falsified. The use of zk-SNARKs also means that the authentication process does not require revealing the UAV’s private information, which is crucial in sensitive operations, thereby ensuring that the authenticity of the UAV can be verified without compromising its operational security.

### 6.2. Challenges

Despite the promising potential of using blockchain technology and zk-SNARKs to enhance the security and privacy of UAV systems, several limitations need to be addressed. One major limitation is the computational overhead associated with generating and verifying zk-SNARK proofs. These cryptographic operations are computationally intensive, which can be a significant constraint for UAVs with limited processing capabilities and energy resources. As illustrated in Figure 13, the mean value for the power consumption is approximately 58.7 W for a period of 60 s. This could be acceptable regarding the UAV mission. For instance, in a small quad-rotor drone, which usually has a 30 Wh battery, if we assume that each rotor demands approximately 30 W, the sensors and communication systems require 15 W, and the navigation systems require 5 W, then the total power consumption is approximately 198.6 W (including the zk-SNARK operations). Therefore, the total flight time is approximately 30 Wh/198.6 W ≈ 0.151 h ≈ 9.1 min. A flight time of 9.1 min is impractical for most applications.

However, things differentiate, in the case of larger UAVs with higher capabilities such as drones that are commonly used in military operations, or for commercial use. Such UAVs could have batteries of 500 Wh (or more) capacity [54]. In that case, for a quad-rotor drone, if we assume that each rotor demands 100 W, and the rest of the technology demands 30 W in total, then the final power demand is approximately 488.6 W including the zk-SNARK operations. Therefore, the flight time can be estimated to be 500 Wh/488.6 W ≈ 1.023 h ≈ 61.4 min, which could be acceptable depending on the nature of the mission.

Another limitation to consider in the case of public blockchains is the cost of blockchain transactions. Based on the thorough analysis in Section 5 for the transaction costs, it is evident that although blockchain provides a secure and tamper-proof platform for recording and verifying transactions, the associated costs can be prohibitive. Each transaction on a blockchain network, such as Ethereum, incurs a gas fee, which can accumulate rapidly in scenarios involving frequent transactions or multiple UAVs. The financial implications of these costs must be carefully considered as the cost of blockchain operations could hinder the widespread adoption of such solutions in resource-constrained environments. These costs are particularly relevant in the context of UAV operations, where maintaining a balance between security and operational efficiency is crucial.

Finally, the integration of zk-SNARKs and blockchain technology introduces challenges related to latency and scalability, which are critical in real-time UAV operations. The computational intensity involved in generating and verifying zk-SNARK proofs can lead to latency, which is particularly problematic in environments where decisions and actions need to be executed within milliseconds. This delay can significantly undermine the effectiveness of UAVs in mission-critical tasks, such as search and rescue or military operations, where swift responses are essential. Furthermore, as the number of UAVs increases, scalability concerns arise due to the growing volume of transactions that must be processed. While blockchain’s decentralized architecture helps distribute the computational load, the sheer scale of operations in large networks can result in congestion and delays, especially on public blockchains. These bottlenecks could slow down transaction times and increase costs, potentially compromising the system’s performance in large-scale deployments.

### 6.3. Future Work

Looking ahead, the integration of blockchain technology and zk-SNARKs into UAV systems presents numerous exciting possibilities for future research and development. One key area for future work is the optimization of zk-SNARK processes to reduce their computational overhead and make them more feasible for use in UAVs with limited processing capabilities. This could involve testing hardware acceleration methods (GPUs or FPGAs) specifically designed for cryptographic operations. Enhancing these aspects will be critical for enabling, in real-time UAV operations, secure communication and verification.

In addition to optimization efforts, rigorous security and privacy tests will be essential in validating the robustness of zk-SNARKs and blockchain integration. Future research should focus on developing comprehensive testing frameworks that assess the resilience of these technologies against various attack vectors, such as replay attacks, man-in-the-middle attacks, and potential side-channel attacks. By conducting extensive real-world tests, critical issues can be identified, allowing the system to be refined to meet the highest security standards for UAV applications.

Finally, another promising avenue for future research is the exploration of applying these techniques in the case of drone swarms. Situations where multiple UAVs need to prove their authenticity or locations simultaneously could be a challenging task. Since aggregating these proofs in real time can become complex, studies on recursive zk-SNARKs or aggregated proofs, which allow for combining multiple proofs into a compact form, could be a good research path that would help in reducing the computational and communication overhead in such networks. This swarm-based UAV case could also help in the understanding of scalability-related issues that arise. As the number of drones in a swarm increases, the system needs to handle a growing number of proofs efficiently. Again, verification techniques that include recursive zk-SNARKs or aggregated proofs could be explored here. This would allow the system to verify multiple zk-SNARK proofs simultaneously and resolve scalability-related issues to an extent.

## Data Availability

The original contributions presented in the study are included in the article; further inquiries can be directed to the corresponding authors.

## References

[1] Alotaibi E.T., Alqefari S.S., Koubaa A. (2019). Lsar: Multi-uav collaboration for search and rescue missions. IEEE Access.

[2] Ramachandran A., Sangaiah A.K. (2021). A review on object detection in unmanned aerial vehicle surveillance. Int. J. Cogn. Comput. Eng..

[3] Boccardo P., Chiabrando F., Dutto F., Tonolo F.G., Lingua A. (2015). UAV Deployment Exercise for Mapping Purposes: Evaluation of Emergency Response Applications. Sensors.

[4] Kim J., Kim S., Ju C., Son H. (2019). I Unmanned aerial vehicles in agriculture: A review of perspective of platform, control, and applications. IEEE Access.

[5] Fernández-Caramés T.M., Blanco-Novoa O., Froiz-Míguez I., Fraga-Lamas P. (2019). Towards an Autonomous Industry 4.0 Warehouse: A UAV and Blockchain-Based System for Inventory and Traceability Applications in Big Data-Driven Supply Chain Management. Sensors.

[6] Zhan P., Yu K., Swindlehurst A.L. (2011). Wireless relay communications with unmanned aerial vehicles: Performance and optimization. IEEE Trans. Aerosp. Electron. Syst..

[7] Khan N.A., Jhanjhi N.Z., Brohi S.N., Nayyar A. (2020). Emerging use of UAV’s: Secure communication protocol issues and challenges. Drones in Smart-Cities.

[8] Allouch A., Cheikhrouhou O., Koubâa A., Khalgui M., Abbes T. MAVSec: Securing the MAVLink protocol for ardupilot/PX4 unmanned aerial systems. Proceedings of the 15th International Wireless Communications & Mobile Computing Conference (IWCMC).

[9] Ko Y., Kim J., Duguma D.G., Astillo P.V., You I., Pau G. (2021). Drone Secure Communication Protocol for Future Sensitive Applications in Military Zone. Sensors.

[10] Alladi T., Chamola V., Sahu N., Guizani M. (2020). Applications of blockchain in unmanned aerial vehicles: A review. Veh. Commun..

[11] Hafeez S., Khan A.R., Al-Quraan M., Mohjazi L., Zoha A., Imran M.A., Sun Y. (2023). Blockchain-assisted UAV communication systems: A comprehensive survey. IEEE Open J. Veh. Technol..

[12] Hafeez M., Javaid N., Khan A., Din I.U. (2020). Blockchain-Based Authentication and Authorization Mechanisms for UAVs: A Survey. IEEE Access.

[13] Morais E., Koens T., Van Wijk C., Koren A. (2019). A survey on zero knowledge range proofs and applications. SN Appl. Sci..

[14] Wan Z., Zhou Y., Ren K. (2022). Zk-AuthFeed: Protecting data feed to smart contracts with authenticated zero knowledge proof. IEEE Trans. Dependable Secur. Comput..

[15] Ernstberger J., Zhang C., Ciprian L., Jovanovic P., Steinhorst S. (2024). Zero-Knowledge Location Privacy via Accurate Floating Point SNARKs. arXiv.

[16] Eberhardt J., Tai S. ZoKrates—Scalable Privacy-Preserving Off-Chain Computations. Proceedings of the IEEE International Conference on Internet of Things (iThings) and IEEE Green Computing and Communications (GreenCom) and IEEE Cyber, Physical and Social Computing (CPSCom) and IEEE Smart Data (SmartData).

[17] Sepolia Resources. https://sepolia.dev/.

[18] Allouch A., Cheikhrouhou O., Koubâa A., Toumi K., Khalgui M., Nguyen G.T. (2021). UTM-Chain: Blockchain-Based Secure Unmanned Traffic Management for Internet of Drones. Sensors.

[19] Aloqaily M., Bouachir O., Boukerche A., Ridhawi I.A. (2021). Design Guidelines for Blockchain-Assisted 5G-UAV Networks. IEEE Netw..

[20] Nguyen T., Katila R., Gia T.N. (2023). An advanced Internet-of-Drones System with Blockchain for improving quality of service of Search and Rescue: A feasibility study. Future Gener. Comput. Syst..

[21] Alsamhi S.H., Shvetsov A.V., Shvetsova S.V., Hawbani A., Guizani M., Alhartomi M.A., Ma O. (2023). Blockchain-Empowered Security and Energy Efficiency of Drone Swarm Consensus for Environment Exploration. IEEE Trans. Green Commun. Netw..

[22] Koulianos A., Litke A. (2023). Blockchain Technology for Secure Communication and Formation Control in Smart Drone Swarms. Future Internet.

[23] Dawaliby S., Aberkane A., Bradai A. Blockchain-based IoT platform for autonomous drone operations management. Proceedings of the 2nd ACM MobiCom Workshop on Drone Assisted Wireless Communications for 5G and Beyond.

[24] Singh M.P., Singh A., Aujla G.S., Singh B.R., Jindal A. Referenced Blockchain Approach for Road Traffic Monitoring in a Smart City using Internet of Drones. Proceedings of the ICC 2022—IEEE International Conference on Communications.

[25] Khan A.A., Laghari A.A., Gadekallu T.R., Shaikh Z.A., Javed A.R., Rashid M., Estrela V.V., Mikhaylov A. (2022). A drone-based data management and optimization using metaheuristic algorithms and blockchain smart contracts in a secure fog environment. Comput. Electr. Eng..

[26] Son S., Kwon D., Lee S., Jeon Y., Das A.K., Park Y. (2023). Design of Secure and Lightweight Authentication Scheme for UAV-Enabled Intelligent Transportation Systems Using Blockchain and PUF. IEEE Access.

[27] Xu R., Chang Z., Zhang X., Hämäläinen T. (2024). Blockchain-Based Resource Trading in Multi-UAV Edge Computing System. IEEE Internet Things J..

[28] Andola N., Raghav, Yadav V.K., Venkatesan S., Verma S. (2021). SpyChain: A lightweight blockchain for authentication and anonymous authorization in IoD. Wirel. Pers. Commun..

[29] Pan H., Wang Y., Wang W., Cao P., Ye F., Wu Q. (2024). Privacy-preserving location authentication for low-altitude UAVs: A blockchain-based approach. Secur. Saf..

[30] Panait A.E., Olimid R.F. On using zk-SNARKs and zk-STARKs in blockchain-based identity management. Proceedings of the Innovative Security Solutions for Information Technology and Communications: 13th International Conference (SecITC).

[31] Kosba A., Miller A., Shi E., Wen Z., Papamanthou C. Hawk: The Blockchain Model of Cryptography and Privacy-Preserving Smart Contracts. Proceedings of the IEEE Symposium on Security and Privacy (SP).

[32] Zheng Z., Xie S., Dai H.N., Chen X., Wang H. (2018). Blockchain challenges and opportunities: A survey. Int. J. Web Grid Serv..

[33] Bodkhe U., Tanwar S., Parekh K., Khanpara P., Tyagi S., Kumar N., Alazab M. (2020). Blockchain for industry 4.0: A comprehensive review. IEEE Access.

[34] Xu M., Chen X., Kou G. (2019). A systematic review of blockchain. Financ. Innov..

[35] Javaid M., Haleem A., Singh R.P., Suman R., Khan S. (2022). A review of Blockchain Technology applications for financial services. BenchCouncil Trans. Benchmarks Stand. Eval..

[36] Esmaeilian B., Sarkis J., Lewis K., Behdad S. (2020). Blockchain for the future of sustainable supply chain management in Industry 4.0. Resour. Conserv. Recycl..

[37] Tanwar S., Parekh K., Evans R. (2020). Blockchain-based electronic healthcare record system for healthcare 4.0 applications. J. Inf. Secur. Appl..

[38] Lin J., Long W., Zhang A., Chai Y. (2020). Blockchain and IoT-based architecture design for intellectual property protection. Int. J. Crowd Sci..

[39] Wei Q., Li B., Chang W., Jia Z., Shen Z., Shao Z. (2020). A survey of blockchain data management systems. ACM Trans. Embed. Comput. Syst. (TECS).

[40] Komalavalli C., Saxena D., Laroiya C. (2020). Overview of blockchain technology concepts. Handbook of Research on Blockchain Technology.

[41] Dotan M., Pignolet Y.A., Schmid S., Tochner S., Zohar A. (2021). Survey on blockchain networking: Context, state-of-the-art, challenges. ACM Comput. Surv. (CSUR).

[42] Lashkari B., Musilek P. (2021). A comprehensive review of blockchain consensus mechanisms. IEEE Access.

[43] Khan S.N., Loukil F., Ghedira-Guegan C., Benkhelifa E., Bani-Hani A. (2021). Blockchain smart contracts: Applications, challenges, and future trends. Peer-to-Peer Netw. Appl..

[44] Islam M.R., Rahman M.M., Mahmud M., Rahman M.A., Mohamad M.H.S. A review on blockchain security issues and challenges. Proceedings of the IEEE 12th Control and System Graduate Research Colloquium (ICSGRC).

[45] Fiege U., Fiat A., Shamir A. Zero knowledge proofs of identity. Proceedings of the Nineteenth Annual ACM Symposium on Theory of Computing (STOC87).

[46] Goldwasser S., Micali S., Rackoff C., Goldreich O. (2019). The knowledge complexity of interactive proof-systems. Providing Sound Foundations for Cryptography.

[47] Chen T., Lu H., Kunpittaya T., Luo A. (2022). A review of zk-SNARKs. arXiv.

[48] Parno B., Howell J., Gentry C., Raykova M. Pinocchio: Nearly Practical Verifiable Computation. Proceedings of the IEEE Symposium on Security and Privacy (SP).

[49] Chiesa A., Hu Y., Maller M., Mishra P., Vesely N., Ward N. Marlin: Preprocessing zkSNARKs with universal and updatable SRS. Proceedings of the Advances in Cryptology–EUROCRYPT: 39th Annual International Conference on the Theory and Applications of Cryptographic Techniques.

[50] Groth J. On the size of pairing-based non-interactive arguments. Proceedings of the Advances in Cryptology–EUROCRYPT: 35th Annual International Conference on the Theory and Applications of Cryptographic Techniques.

[51] https://remix.ethereum.org.

[52] Baghery K., Pindado Z., Ràfols C. Simulation extractable versions of Groth’s zk-SNARK revisited. Proceedings of the 19th International Conference on Cryptology and Network Security.

[53] Garg S., Goel A., Jain A., Policharla G.V., Sekar S. zkSaaS: Zero-KnowledgeSNARKs as a Service. Proceedings of the 32nd USENIX Security Symposium.

[54] Park S., Zhang L., Chakraborty S. Battery assignment and scheduling for drone delivery businesses. Proceedings of the IEEE/ACM International Symposium on Low Power Electronics and Design (ISLPED).

